# In Vivo Imaging Sheds Light on the Susceptibility and Permissivity of *Carassius auratus* to Cyprinid Herpesvirus 2 According to Developmental Stage

**DOI:** 10.3390/v15081746

**Published:** 2023-08-15

**Authors:** Bo He, Arun Sridhar, Cindy Streiff, Caroline Deketelaere, Haiyan Zhang, Yuan Gao, Yunlong Hu, Sebastien Pirotte, Natacha Delrez, Andrew J. Davison, Owen Donohoe, Alain F. C. Vanderplasschen

**Affiliations:** 1 Immunology-Vaccinology, Department of Infectious and Parasitic Diseases, Fundamental and Applied Research for Animals & Health (FARAH), Faculty of Veterinary Medicine, University of Liège, B-4000 Liège, Belgium; bo.he@doct.uliege.be (B.H.); arun.Sridhar@uliege.be (A.S.); cindy.streiff@uliege.be (C.S.); cdeketelaere@uliege.be (C.D.); haiyanzhang199104@163.com (H.Z.); gaoyuan@bio-ss.net (Y.G.); yunlong.hu@uliege.be (Y.H.); spirotte@student.uliege.be (S.P.); natacha.delrez@gmail.com (N.D.); owen.donohoe@uliege.be (O.D.); 2 MRC-University of Glasgow Centre for Virus Research, Glasgow G61 1QH, UK; andrew.davison@glasgow.ac.uk; 3 Bioscience Research Institute, Technological University of the Shannon, Athlone N37 HD68, Co. Westmeath, Ireland

**Keywords:** virology, Cyprinid Herpesvirus 2, *Alloherpesviridae*, Cyprinivirus, *Carassius auratus*, aquaculture, pathogenesis, portal of entry, recombinant viruses, in vivo imaging

## Abstract

Cyprinid herpesvirus 2 (CyHV-2) is a virus that causes mass mortality in economically important *Carassius* spp. However, there have been no comprehensive studies into host susceptibility or permissivity with respect to developmental stage, and the major portal of viral entry into the host is still unclear. To help bridge these knowledge gaps, we developed the first ever recombinant strain of CyHV-2 expressing bioluminescent and fluorescent reporter genes. Infection of *Carassius auratus* hosts with this recombinant by immersion facilitated the exploitation of various in vivo imaging techniques to establish the spatiotemporal aspects of CyHV-2 replication at larval, juvenile, and adult developmental stages. While less susceptible than later developmental stages, larvae were most permissive to CyHV-2 replication, leading to rapid systemic infection and high mortality. Permissivity to CyHV-2 decreased with advancing development, with adults being the least permissive and, thus, also exhibiting the least mortality. Across all developmental stages, the skin was the most susceptible and permissive organ to infection at the earliest sampling points post-infection, indicating that it represents the major portal of entry into these hosts. Collectively these findings provide important fundamental insights into CyHV-2 pathogenesis and epidemiology in *Carassius auratus* with high relevance to other related economically important virus-host models.

## 1. Introduction

The goldfish (*Carassius auratus*) is a freshwater teleost species belonging to the Carassius genus within the *Cyprinidae* family. It has a rich history of domestication in China, spanning approximately 1000 years, and has emerged as a highly popular ornamental fish species. Goldfish were introduced to Japan and Europe during the early 17th century and subsequently reached North America around 1850 CE, where they gained significant popularity. Breeders and enthusiasts worldwide have successfully produced over 180 variants of goldfish, which continue to be highly sought after due to their remarkable morphological diversity and colour variations [1,2]. Beyond their ornamental value, the varied morphologies of goldfish strains have captured the interest of biologists and has been utilized as an experimental organism in the study of developmental biology and even as models for diseases including cardiac and retinal conditions [1,3,4].

In addition, goldfish have also been utilized as models to study infectious diseases, in particular prokaryotic pathogens [5,6,7]. Like other cultured fish species, goldfish have highly distinct, well studied developmental stages, which may make them ideal models for assessing the impact of the developmental stage on susceptibility to infectious disease. Briefly, after egg fertilization, goldfish embryo development typically lasts three days, after which larvae hatch and exhibit vigorous swimming and feeding behaviors [6,7]. By 45 to 49 days post-fertilization (dpf), larvae acquire an adult shape, lose their fin fold, and transition into the juvenile stage. Finally, possibly up to one-year post-fertilization, goldfish reach a body length exceeding 5 cm and begin to develop external reproductive characteristics, coinciding with the commencement of the adult stage [8,9]. Studying the impact of developmental stage on susceptibility and permissivity to infectious disease may be of particular importance to the goldfish aquaculture industry, in terms of better understanding and assessment of risk at various stages of the rearing process. Findings gained from goldfish models may be of relevance to other closely related economically important *Carassius* spp., which may be susceptible to many of the same pathogens, and account for much larger portions of global aquaculture output [10].

In addition to bacterial, fungal, and parasitic pathogens, diseases caused by viral infection represent a major cause of economic loss within the aquaculture industry [11,12,13]. Indeed, viral disease is also a serious problem in the goldfish aquaculture sector. The most problematic and frequently isolated virus of goldfish is cyprinid herpesvirus 2 (CyHV-2), which causes mass mortality among goldfish populations. CyHV-2 is a member of the genus *Cyprinivirus*, in the family *Alloherpesviridae*. Two closely related viruses within the *Cyprinivirus* genus, cyprinid herpesvirus 1 (CyHV-1) and cyprinid herpesvirus 3 (CyHV-3) also infect cyprinid fish species, such as common carp (*Cyprinus carpio*).

The first known CyHV-2 outbreaks occurred in goldfish in Japan in 1992 and 1993 and were described in 1995 [14]. Further major outbreaks in goldfish were reported in 1998 in the western United states [15] and a year later in Taiwan [16]. In the following years, disease caused by CyHV-2 also reported among goldfish in many other regions including Australia [17], UK [18], India [19], Switzerland [20], Germany [21], France [22], Netherlands [23], Turkey [24] and Poland [25]. While initial reports were restricted to goldfish, outbreaks were also observed among populations of closely related fish species, Crucian carp (*Carassius carassius*) and Gibel carp (*Carassius gibelio*) [26,27,28,29,30], which both represent much larger aquaculture production sectors relative to goldfish [10,31].

However, like many related fish viruses, systemic replication of CyHV-2 in hosts is dependent on water temperature. Indeed, outbreaks of CyHV-2 that cause mass mortality are largely restricted to periods when water temperatures are within a 15–25 °C range [18,30,32]. During infections, the main external clinical signs associated with CyHV-2 infection are lethargy, anorexia, pale skin accompanied by hemorrhaging and blisters on the fins, pale gills, and elevated respiratory efforts. Infected fish also exhibit internal lesions and pale and swollen kidney and spleen (extensively reviewed elsewhere [30]). CyHV-2 may also establish persistent infections [33], which may re-emerge in response to stress, for example changes in water temperature or transportation processes [30], and during periods of immunosuppression [34]. While horizontal transmission may be the dominant mode of CyHV-2 between fish, evidence indicates that vertical transmission (from parents to offspring) may also occur [35,36].

There is a large and growing body of scientific literature describing investigations into the aetiology, diagnosis, transmission, and prevention of CyHV-2 [14,15,30]. However, to date, there is only limited knowledge of key aspects of CyHV-2 pathogenesis (such as the portal of entry of the virus into its host), and no systematic comparison of the differences in susceptibility and permissivity between goldfish developmental stages. On the later point, given that CyHV-2 was initially identified as being mainly a cause of disease in juvenile goldfish [14,15,16], this may indicate that earlier goldfish developmental stages are more susceptible [30].

In the present study, we generated and characterized the first ever recombinant strain of CyHV-2 expressing both luciferase 2 (Luc) and copepod GFP (copGFP) reporter genes, which we refer to in this study as the LucGFP strain. Using this recombinant strain, we focused on elucidating two key fundamental aspects of CyHV-2 pathogenesis in goldfish: (1) the relative susceptibility (the ability to support CyHV-2 entry) and permissivity (the ability to support CyHV-2 replication) across different developmental stages and (2) the route of viral entry into the host. Closing these knowledge gaps provides important insights into CyHV-2 pathogenesis.

## 2. Materials and Methods

### 2.1. Cells and Viruses

The RyuF-2 cell line, derived from the caudal fin of Ryukin goldfish, was used to propagate the virus in this study [37]. Cells were cultured at 25 °C using Medium 199 (HEPES buffered; Sigma, Kawasaki, Japan) supplemented with 10% fetal bovine serum (FBS; Gibco, Life Technologies, Carlsbad, CA, USA), penicillin (100 U/mL), and streptomycin (100 μg/mL). The CyHV-2 YC-01 strain (GenBank: MN593216.1) was isolated from diseased gibel carp [38] and kindly provided by Prof. Lu (Shanghai Ocean University, Pudong, Shanghai, China). CyHV-2 was cultured in RyuF-2 cells at 25 °C using the same cell culture media described above. After the widespread CPE was observed, viral culture media were collected and centrifuged at 2000× *g* for 20 min (min) (Allegra X-15R Centrifuge, Beckman, Brea, CA, USA) to remove cell debris. The resulting viral supernatant was stored at −80 °C. Titers of infectious CyHV-2 particles were determined by triplicate plaque assays conducted in 6-well plates. Briefly, a series of 10-fold dilutions were made from viral supernatant, using serum free cell culture medium as diluent. A total of 1 mL of each dilution was applied to each well containing fresh RyuF-2 cells, 18–24 h (hour) post-seeding (80–100% confluent), and incubated for 2 h at 25 °C. Subsequently, the initial inoculum was removed from each well and replaced with 3 mL complete cell culture media with carboxymethylcellulose (CMC) (final concentration 2% *w*/*v*) and incubated for 4 days at 25 °C. Plaques were visualized using an epifluorescence microscope (Leica DM2000), either indirectly using fluorescent immunostaining or directly using a virally expressed fluorescent reporter. Dilutions exhibiting 20–200 plaques were used to estimate titers.

### 2.2. Production of the LucGFP Recombinant Strain of CyHV-2 by Recombination in Eukaryotic Cells

**Figure 1:**
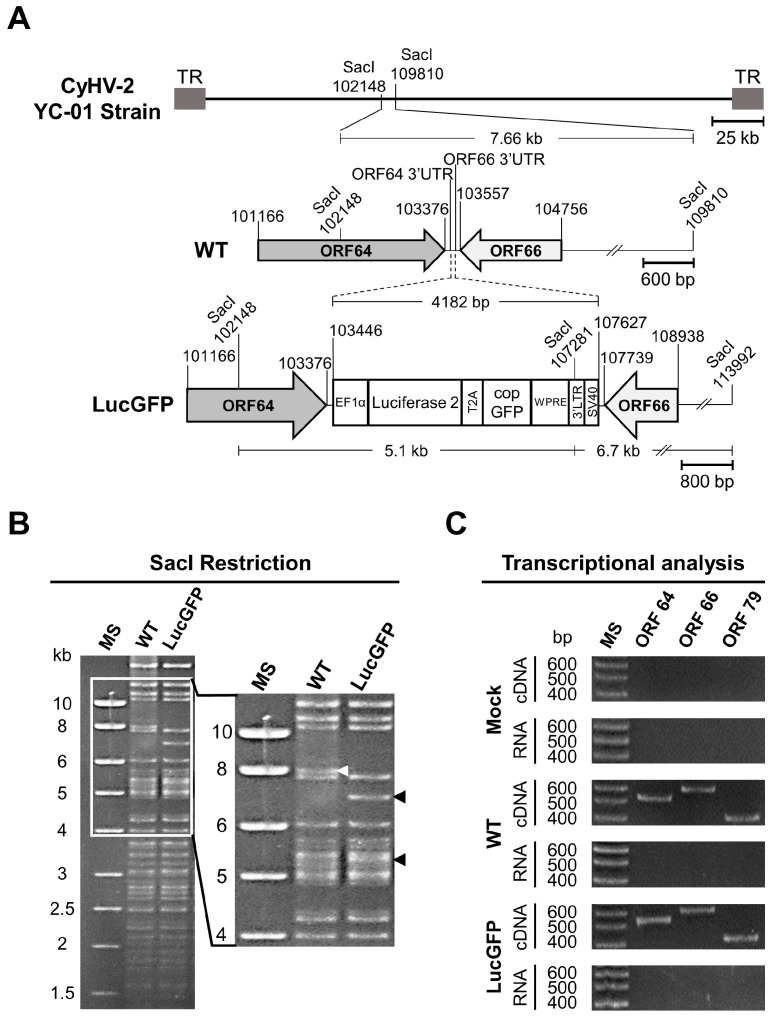
**Production of the CyHV-2 LucGFP recombinant strain.** (**A**) Schematic representation of the genome structure of the recombinant produced. The genome of the CyHV-2 YC-01 strain, flanked by two terminal repeats (LTR and RTR), is shown at the top. A bicistronic reporter expression cassette was inserted into the non-coding ORF64-ORF66 intergenic region. SacI restriction sites and genome coordinates are marked. (**B**) Genomic analysis of CyHV-2 strains. The genomes of the WT and LucGFP strains were analyzed by SacI RFLP analysis. The white arrowhead indicates the fragment (7.66 kb) of the WT strain genome containing the insertion site. The black arrowheads indicate two new fragments (5.1 and 6.7 kb) that were generated due to the insertion of the LucGFP cassette. The ladder indicates the approximate sizes of each fragment. (**C**) Transcriptional analysis of genes flanking the insertion site of the transgene. Using reverse transcriptase polymerase chain reaction (RT-PCR), transcription of ORF64 and ORF66 was compared between the LucGFP strain and WT strain during viral replication in vitro. Both ORF64 (546 bp) and ORF66 (610 bp) transcripts were detected in RNA from WT and LucGFP infected cells, indicating that flanking genes were not impacted by the reporter gene insertion. Importantly, the absence of a detectable product in the RT-negative controls (RNA, bottom row) indicated that results were not the result of residual genomic DNA contaminants. Marker sizes (MS) are indicated on the left. ORF79 (CyHV-2, DNA polymerase gene) was used as a loading control, as it is located in a distant part of the CyHV-2 genome and, thus, should not be impacted by the reporter gene insertion between ORF64 and ORF66.

The CyHV-2 LucGFP strain was produced by homologous recombination in RyuF-2 cells (Figure 1). The LucGFP cassette consisted of an EF1α promoter driving the transcription of a bicistronic mRNA encoding Luc and copGFP proteins linked by a T2A peptide. The LucGFP cassette was inserted in the intergenic region between ORF64 and ORF66 of CyHV-2 genome to produce the LucGFP recombinant strain. This targeted insertion was achieved by initially ligating the LucGFP cassette into a pGEMT vector, flanked by 500-bp sequences corresponding to the end and the beginning of CyHV-2 ORF64 and ORF66, respectively, to create the pGEMT LucGFP vector. To create the pGEMT LucGFP vector, target fragments were first amplified using the Phusion High-Fidelity PCR Kit (New England Biolabs, Ipswich, MA, USA), with overlaps for assembly incorporated into the primers. All corresponding templates and primers are listed in Table 1. The amplified fragments and an empty pGEMT vector were assembled together using the NEBuilder HiFi DNA Assembly Kit (New England Biolabs), with the ligation product transformed into competent bacterial strains. The pGEMT LucGFP vector was purified using the NucleoSpin Plasmid Mini Kit (Macherey Nage, Düren, Germany). One day after transfection with the pGEMT LucGFP vector, RyuF-2 cells were infected with the CyHV-2 YC-01 strain (WT parental strain) at the multiplicity of infection (MOI) of 0.1 plaque forming units (pfu)/cell. After 4 days, the supernatant was collected and used to make a series of 10-fold dilutions in serum free cell culture media, which were then used to infect fresh RyuF-2 cells. Cells were infected in the same way as described for viral titration above. Isolated viral plaques expressing copGFP were picked and subcultured until 100% of plaques in subsequent sub-cultures were observed to express the reporter gene. Plaques were viewed using an epifluorescence microscope (Leica DM2000).

### 2.3. Genetic Characterization of the CyHV-2 LucGFP Recombinant

The genotype of the recombinant strain was confirmed by SacI restriction fragment length polymorphism (RFLP) analysis. Briefly, 3 µg of genomic DNA was digested using SacI (New England Biolabs). Digested DNA was run in 0.8% agarose gel at 60 V for 18 h. Full-length genome sequencing was performed as described previously [39].

### 2.4. Transcription Analysis

RyuF-2 cells were mock-infected with cell culture media or infected with CyHV-2 at an MOI of 0.1 pfu/cell. RNA was isolated 24 h post-infection (hpi) using the NucleoSpin RNA Mini Kit (Macherey-Nagel), and residual DNA was removed using the TURBO DNA-free Kit (Invitrogen, Waltham, MA, USA). Reverse transcription reactions were performed on 5 μg of RNA using Superscript III Reverse Transcriptase with oligo (dT) primers (Invitrogen). Finally, ORF64, ORF66 and ORF79 (CyHV-2 DNA polymerase) were amplified using the Phusion High-Fidelity DNA polymerase kit (New England Biolabs) with the pairs of primers described in Table 1.

### 2.5. Polyclonal Antibody Production

CyHV-2-specific rabbit polyclonal antibodies (pAbs) were produced following a 70-day rabbit immunization protocol. Viral supernatant was prepared from infected RyuF-2 cells as described above. Virions were then pelleted by centrifugation of 100,000× *g* for 2 h using an ultracentrifuge (Optima LE-80K Ultracentrifuge, Beckman). The supernatant was removed, and the virion pellet was resuspended in 1 mL PBS to create a CyHV-2 antigen suspension. The total protein concentration of the CyHV-2 antigen suspension was measured using a Pierce BCA assay kit (Thermo Fisher, Waltham, MA, USA), after which it was diluted accordingly in PBS. Diluted CyHV-2 antigen was mixed thoroughly with adjuvant (50:50 *v*/*v*) using a TissueLyser (QIAGEN) in order to obtain an antigen-adjuvant emulsion solution. This emulsion was administered to specific pathogen-free rabbits via a series of subcutaneous injections (100 ng of total protein per injection). The primary immunization consisted of 500 μL of antigen and 500 μL of Freund’s complete adjuvant administered at 5 different injection sites. All boosters consisted of 500 μL antigen and 500 μL incomplete Freund’s adjuvant emulsion and were also administered at 5 different injection sites. Boosters were administered at two, four, and six weeks after the primary immunization. Blood was collected two weeks after the final booster and incubated at 37 °C for 1–2 h before being transferred to 4 °C overnight. The samples were subsequently centrifuged at 900× *g* for 20 min, and serum was collected and stored at −80 °C.

### 2.6. Indirect Immunofluorescence Staining

RyuF-2 cells in 6-well plates (mock-infected with cell culture media or infected with CyHV-2) were fixed with PBS containing 4% (*w*/*v*) paraformaldehyde at 4 °C for 15 min and then 20 °C for 15 min. After washing with PBS, samples were permeabilized in PBS containing 0.1% (*v*/*v*) NP-40 at 37 °C for 15 min. Immunofluorescent staining (incubation and washes) was performed in PBS containing 10% FCS (*v*/*v*). The primary antibody, consisting of rabbit anti-CyHV-2 pAbs, was diluted 1:2000 in FCS-PBS and incubated on fixed and permeabilized cells at 37 °C for 2 h. The secondary antibody, Alexa Fluor 568 goat anti-rabbit immunoglobulin G (H + L) (2 μg/mL; Molecular Probes, Invitrogen), was diluted 1:1000 in 10% (*v*/*v*) FCS-PBS and incubated at 37 °C for 30 min. After washing, stained cells were treated using Prolong Gold Antifade Reagent (Invitrogen). Cells were viewed using an epifluorescence (Leica DM2000) or confocal (Nikon A1R) microscope.

### 2.7. Viral Growth Assay

CyHV-2 was diluted in serum-free media to give a MOI of 0.01 pfu/cell in each well. Triplicate cultures of RyuF-2 cells grown in 6-well plates were infected with 1 mL viral supernatant. After an incubation period of 2 h, the cells were washed with PBS and overlaid with complete cell culture medium. At 2-, 4-, and 6-days post-infection (dpi), both viral supernatant and infected cells were collected from each well, combined, and stored at −80 °C for one week. These samples were thawed and analyzed by viral titration using triplicate plaque assays in RyuF-2 cells, as described above.

### 2.8. Viral Plaque Assay

RyuF-2 cells were cultured in six-well plates and inoculated with 100 pfu/well of CyHV-2 for 2 h, and then overlaid with culture medium supplemented with CMC (2% *w*/*v*). At 3, 4, 5, and 6 dpi, viral plaques were visualized by indirect immunofluorescent staining. After the final wash with PBS, 20 randomly selected individual plaques were then imaged using a Nikon A1R confocal microscope, and areas were measured using ImageJ software (Version 1.53) [40].

### 2.9. Fish

Three different development stages of Shubunkin goldfish (*Carassius auratus*) were used in the present study. (i) Mature shubunkin goldfish were obtained from an accredited commercial company (Ruinemans Aquarium, Montfoort, The Netherlands). Microbiological, parasitic, and clinical examinations were conducted immediately after arrival in the animal facility and then monthly to monitor fish health. Adult goldfish from the colony were determined to be free of CyHV-2 based on polymerase chain reaction (PCR) analysis of kidney homogenate. Fish were maintained in 60 L freshwater recirculation tanks at 25 °C until used for breeding or transferred to L2 facilities for infection experiments. (ii) Shubunkin goldfish larvae (4 dpf) were produced using standard artificial reproduction methods applied previously [41]. The larvae were hatched in Zoug bottles, then transferred to 60 L freshwater recirculation tanks maintained at 25 °C after hatching until being transferred to a L2 laboratory setting for infection experiments at 4 dpf. (iii) Juvenile shubunkin goldfish (75 dpf) were generated through rearing of the larvae mentioned above, and were maintained in 60 L freshwater recirculation tanks at 25 °C.

### 2.10. Inoculation of Fish with CyHV-2

Different modes of inoculation were used depending on the developmental stage. In all cases, viral inoculum was thawed and used immediately. (i) Inoculation of adult goldfish by intraperitoneal (IP) injection: Shubunkin adult goldfish (average weight 3.5 ± 0.4 g, 8 months old) were first anesthetized by immersion in water containing benzocaine (25 mg/L) and weighed. The virus was diluted in serum free cell culture medium (final concentration = 5 × 10^5^ pfu/mL) and injected intraperitoneally using a 300 μL insulin syringe (BD Micro-Fine), with each fish receiving a dose of 10^4^ pfu/g (total body weight). After injection, the fish were placed in an aerated recovery bath for 10 min and returned to the tank. (ii) Inoculation of larvae by immersion in infectious water: Goldfish larvae (4 dpf, average length = 5.2 ± 0.1 mm) were infected by immersion in E3 medium containing virus. Briefly, larvae were transferred to a single petri dish (1 larvae per mL of E3 media) and CyHV-2 was added (final concentration = 1 × 10^5^ pfu/mL) followed by gentle mixing. The larvae were incubated at 25 °C for 2 h, and subsequently transferred to new petri dishes containing 50 mL of E3 medium. (iii) Inoculation of juvenile fish by immersion in infectious water: Juvenile shubunkin goldfish (75 dpf, average length = 2.3 ± 0.3 cm) were immersed in water containing CyHV-2 (final concentration = 1 × 10^5^ pfu/mL) for 2 h under constant aeration (20 fish/L), and then moved to a separate virus free tank. (iv) Inoculation of adult fish by immersion in infectious water: Adult shubunkin goldfish (1.5 years old, average weight = 12 ± 3.7 g) were immersed in water containing CyHV-2 (final concentration = 1 × 10^5^ pfu/mL) for 2 h under constant aeration (10 fish/L), and then moved to a 60 L tank. In all experiments, mock-infected fish were also prepared by the substitution of viral inoculum with serum-free cell culture medium.

### 2.11. Scoring of Morbidity and Mortality

The percentage of fish expressing clinical signs was determined by daily examination of each tank. The percentage of morbid fish was defined as the proportion of fish in each tank expressing one or several of the following clinical signs: hyperemia of cephalic skin, hyperemia of the gills, hyperemia of the skin covering the body or the fins, multifocal skin lesions, or abnormal swimming. Mortality was recorded daily, and the survival rate was calculated at the end of the experiment.

### 2.12. Bioluminescent Imaging

Luminescent emission from firefly (*Photinus pyralis*) luciferase was imaged using an in vivo imaging system (IVIS) (Spectrum, Perkin Elmer, Rowville, Australia) as described previously [42]. For in vivo analysis, adult and juvenile fish were anesthetized with benzocaine (25 mg/L), in aerated water. Once anesthetized, D-luciferin (Caliper LifeSciences) was administered by IP injection (150 mg/kg of body weight). Fish were kept for 15 min in aerated water before bioluminescence was measured. Larvae were anesthetized by transferring to a new petri dish with 0.2 mg/mL tricaine (MS222, Sigma-Aldrich, St. Louis, MI, USA) in E3 medium (1 fish/mL). This E3 also contained methylcellulose (2%) in order to reduce drifting of the larvae during subsequent procedures. D-luciferin (150 μg/mL) was administered to larvae by pericardial microinjection (3 nL/fish). After 5 min incubation, larvae were imaged by IVIS. For all developmental stages, the bioluminescent signal originating from the skin was imaged first. Each fish was analyzed lying on its left and right side, except larvae, which were acquired for a single position. After euthanasia of fish with benzocaine (250 mg/L), organs (adult: gill, gut, heart, spleen, and kidney; juvenile: gill and gut; larvae: not applicable) were dissected and bioluminescence signals were measured by IVIS separately from the body. Adult images presented in this study were acquired using a field view of C while juvenile and larvae were acquired using the field view B. Other settings, including binning factor = 8, f/stop = 1, and maximum auto-exposure time of 1 min, were constant for all developmental stages. Data were analyzed using the Living Image software (v3.2) (Perkin Elmer). Each region of interest (ROI) was drawn manually by tracing the organs or body outlines, and the average radiance (p/sec/cm^2^/sr) was taken as the final measure of the bioluminescence emitted over the ROI. Images were annotated to show the average radiance signal according to location in the subject, using a colour scale ranging from violet (lowest signal) to red (highest signal). For the skin, the average radiance (individual values, mean + standard deviation or SD) was measured on both sides of the body (except for larvae), and the results for individual fish were expressed as the mean of both sides. For each fish, corresponding average radiance values from gills (mean of left and right gills) and internal organs (gut, heart, spleen, and kidney), were measured and presented separately. The cut-off for positivity (dotted line in all related figures) consisted of the mean +3 SD of the values obtained for mock-infected fish (negative control). For measurement of bioluminescence in vitro, cell culture media was replaced with a fresh serum-free media containing D-luciferin (150 μg/mL). After a 10 min incubation at room temperature, bioluminescence signals were acquired using the same IVIS settings as used for adult fish, and the entire well was used as the ROI when establishing the average radiance.

### 2.13. In Vivo Epifluorescence Microscopy

For epifluorescence microscopy analysis of adults (skin and caudal fin), juveniles (eye, gill, skin, and caudal fin), and larvae (whole body), fish were anesthetized as described for IVIS analysis above. Images were captured with a Leica DM2000 epifluorescence microscope using the GFP filter set at 5× or 10× magnification.

### 2.14. Light Sheet Microscopy Imaging

This experimental procedure was adapted from previous methodology used with zebrafish larvae [43,44,45]. Briefly, larvae were infected or mock-infected with the CyHV-2 LucGFP strain by immersion as described previously. After 24 h of incubation, infection was confirmed using epifluorescence microscopy to identify the GFP signal. To stain the nuclei of living cells of skin epidermis, infected larvae were incubated in E3 media containing DRAQ5 (1:1000 dilution) for 30 min. In parallel, immobilizing agarose was prepared by dissolving low-melting agarose (Bio-Rad, Hercules, CA, USA) (final concentration 0.1% *w*/*v*). Once the agarose was dissolved, tricaine was added to the immobilizing agarose solution (0.1% *w*/*v* final concentration). An additional agarose solution consisting of standard high melting point agarose (final concentration 1% *w*/*v*) in E3 was also prepared for sealing the sample chambers. After the DRAQ5 staining was complete, larvae were anesthetized as described above, and transferred to the immobilizing agarose solution containing 0.1% (*w*/*v*) tricaine. Fluorinated ethylene–propylene (FEP) tubing (Bola, S1815-07, refractive index 1.338, inner diameter 2 mm, outer diameter 3 mm) was used as the sample chamber and attached to the end of a capillary. By placing a plunger in the capillary, a single larvae suspended in immobilizing agarose solution was drawn up into FEP tubing (headfirst). The end of the FEP tubing was sealed by drawing up standard high melting point agarose (1% *w*/*v*) which solidified quickly after entry to the FEP tubing. A three-dimensional image was acquired using a Zeiss Light-sheet Z1 10× objective along the *y* axis, pairing 488 nm and 638 nm laser to excite GFP and DRAQ5, respectively. The subsequent data was used to generate a maximum-intensity projection video with ImageJ.

### 2.15. Confocal Microscopy Imaging

DRAQ5-stained larvae were prepared as described for light-sheet microscopy. They were anesthetized in E3 medium containing 0.2 mg/mL of tricaine and transferred to a glass slide with a small droplet of E3 medium. A cover slip with spacers was placed over larvae, providing enough space to accommodate the subject under the coverslip. The slides were analyzed by confocal microscopy (Nikon A1R) using 488 nm and 633 nm lasers to excite GFP and DRAQ5, respectively. A series of Z-stack confocal images were acquired to generate a 3D representation of the area of interest. The acquired images were rendered using Imaris viewer software (v10.0) (Oxford Instruments, Oxfordshire, UK).

### 2.16. Immunohistochemistry

For immunohistochemistry (IHC), tissues were fixed in 4% PAF for 18 h at 4 °C then dipped in 70% (*v*/*v*) ethanol and embedded in paraffin. Immunoperoxidase staining was performed on tissue sections (5 µm) after dewaxing and epitope unmasking in citrate buffer (0.01 M, pH 6.0). Endogenous peroxidases were blocked by incubation in 3% H_2_O_2_ for 15 min at room temperature (RT). After blocking of non-specific binding sites, tissues were incubated for 1 h at 22 °C in the primary antibody (mouse monoclonal turboGFP antibody, OriGene Technologies) diluted 1:500. Sections were then incubated for 1 h at room temperature (RT) with the secondary antibody (Envision anti-mouse + System-HRP, Agilent). Peroxidase was detected with Liquid DAB + Substrate Chromogen System (Agilent). After rinsing with demineralized water, sections were counterstained with Carazzi’s hematoxylin (EMD Millipore, Burlington, MA, USA). Sections of mock-infected fish served as negative controls. Specimens were scanned using a Nanozoomer Digital Pathology 2.0 HT scanner (Hamamatsu Photonics, Shizuoka, Japan) and were analyzed using NDP.view2 Viewer software (Hamamatsu Photonics).

### 2.17. Statistical Analysis

Residuals for each dataset were first tested for normality using the Shapiro–Wilk test (GraphPad Prism v8.0.1) to determine if parametric and non-parametric tests were required. Two-way omnibus tests on data from the viral growth curve and plaque size experiments were conducted using two-way analysis of variance (ANOVA) with multiple comparisons between groups of interest made using the Benjamini–Hochberg (BH) post hoc test. One-way tests on data generated from the in vivo experiments were conducted using the one-way ANOVA test with BH post hoc test for data sets exhibiting normal distribution, or the Kruskal–Wallis Test with Dunn’s post hoc test for data not exhibiting normal distribution. Survival curves were compared using the log-rank test. All tests were implemented in GraphPad prism 8.0. Only significant *p*-values (<0.05) are reported in the results section. Similarly, for the purposes of visual clarity, only significant results from post hoc multiple comparisons are indicated in each corresponding figure, and are represented using the following symbols: * = *p* < 0.05; ** = *p* < 0.01; *** = *p* < 0.001.

### 2.18. Ethics Statement

The experiments, maintenance, and care of fish complied with the guidelines of the European Convention for the Protection of Vertebrate Animals used for Experimental and other Scientific Purposes (CETS No. 123). The animal studies were approved by the local ethics committee of the University of Liège, Belgium (Laboratory accreditation No. 1610008). All efforts were made to minimize suffering.

## 3. Results

The primary aim of this study was to conduct a systematic investigation into the spatiotemporal aspects of CyHV-2 replication using in vivo imaging in goldfish across larvae, juvenile, and adult developmental stages. To maximize the consistency and homogeneity between experimental subject populations at different developmental stages, all the fish used in this study were derived from a single batch, thus maximizing the overall scientific validity of our findings. Specifically, goldfish were selectively bred and raised to reach adulthood, attaining an age of 1.5 years, before being segregated into two distinct schools. One group of fish was subjected to direct infection with CyHV-2, serving as the experimental host population. Simultaneously, another group of fish was utilized solely for breeding purposes, facilitating the generation of offspring. The resulting progeny were further divided into two subgroups: a portion was utilized in experimental infections at the larvae stage, while the remaining individuals were allowed to grow to the juvenile stage for subsequent experimentation.

### 3.1. Generation of the CyHV-2 LucGFP Recombinant Strain

We have previously demonstrated that in vivo imaging is a highly effective methodology for the comprehensive investigation into pathogenesis associated with CyHV-3 [39,41,42,46,47,48,49] which is closely related to CyHV-2 [50,51]. To facilitate in vivo imaging during CyHV-2 experiments, the YC-01 CyHV-2 strain (WT) was selected as the parental strain for generation of a recombinant expressing Luc and copGFP as reporter proteins, which we refer to as the LucGFP strain. The reporter cassette was inserted between ORF64 and ORF66 of the WT CyHV-2 genome, as depicted in Figure 1A. This insertion of the construct resulted in the introduction of a new SacI restriction site at position 107281 of the LucGFP genome, resulting in the expected loss of the 7.66 kb SacI restriction fragment in the parental WT strain and an appearance of two smaller 5.1 KB and 6.7 kb SacI restriction fragments in the LucGFP strain (Figure 1A). The expected pattern was observed in subsequent SacI RFLP analysis (Figure 1B), thus, confirming the correct integration of the reporter cassette, which was also confirmed by whole genome sequencing (data not shown). Furthermore, transcriptomic analysis of ORF64 and ORF66 genes during LucGFP replication in vitro confirmed that the reporter cassette did not prevent the expression of genes adjacent to the insert in the LucGFP strain (Figure 1C).

### 3.2. Comparison of LucGFP Recombinant and WT Strains In Vitro and In Vivo

**Figure 2:**
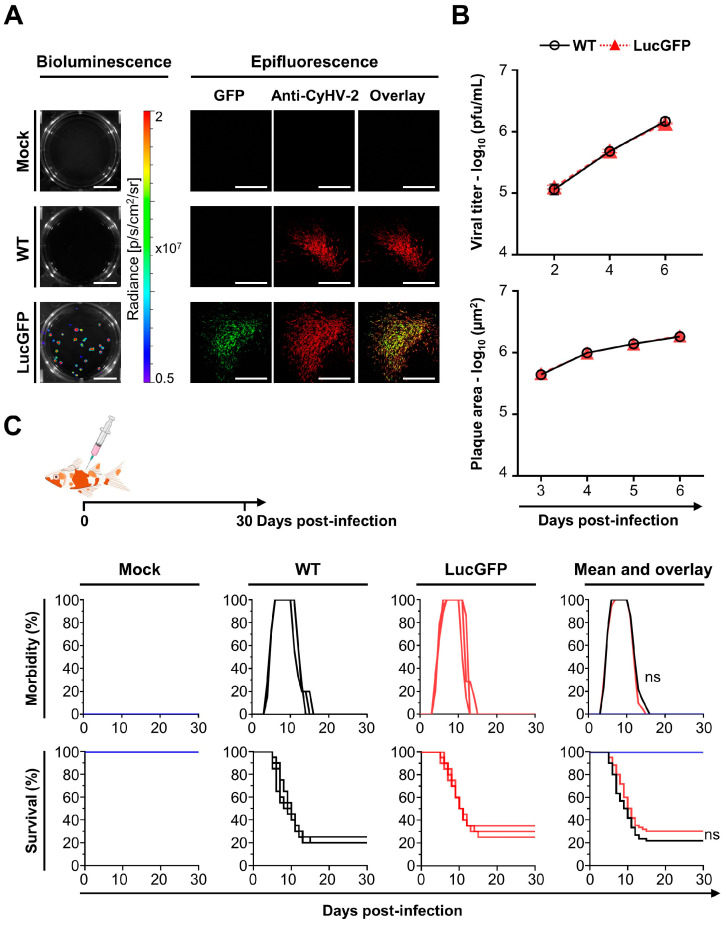
**Phenotypic characterization of CyHV-2 strains.** (**A**) Expression of reporter genes. RyuF-2 cells grown in 6-well plates were infected with the indicated strains and then overlaid with medium containing CMC. At 4 dpi, infected cells were analyzed for bioluminescent and fluorescent signal from reporter genes. The Luc signal was detected using an IVIS system (**left** column, scale bar = 1 cm). The copGFP signal was detected by epifluorescence microscopy (**right** panels, scale bar = 500 μm). Viral plaques were revealed by indirect immunofluorescent staining. (**B**) Comparisons of viral growth in vitro. Viral growth assay (**top** panel). RyuF-2 cells were infected with the indicated strains and the log_10_ value of the titer (pfu/mL) in the supernatant was determined at the indicated dpi. Data represent the mean ± SEM of triplicate measurements. Viral plaque assay (**lower** panel). RyuF-2 cells were infected with the indicated strains, and plaque areas were measured over time. Data represent the mean ± SEM of 20 individual plaques. No significant differences were detected between WT and LucGFP strains. (**C**) Comparison of virulence in vivo. The virulence of the indicated strains was tested in adult Shubunkin goldfish (triplicate groups each consisting of 20 subjects, average weight 3.5 ± 0.4 g, 8 months old). Fish were mock-infected or infected by IP injection with the indicated strains (10^4^ pfu/g). The fish were examined daily for clinical signs of CyHV-2 disease, and fish reaching the endpoints were euthanized. No significant differences were detected between WT and LucGFP strains in the experiments described in (**B**,**C**).

The ability of the LucGFP recombinant to express Luc and copGFP was confirmed in vitro. Briefly, RyuF-2 cells were infected with a 10^−5^ dilution of the original LucGFP and WT stock, and then cultured under the conditions described for plaque assays. At 4 dpi, bioluminescence imaging was performed by IVIS, confirming the expression of Luc in the LucGFP-infected monolayer, with no signal observed in WT and mock-infected cells (Figure 2A, left panel). Replicate plates were examined through indirect immunofluorescent staining and epifluorescence microscopy, confirming both the expression and colocalization of copGFP and viral plaque signals (Figure 2A, right panel). Taken together, these results confirmed the correct expression of both reporter genes by the LucGFP recombinant.

To verify that the insertion of the construct in the LucGFP strain did not have any impact on viral growth in vitro relative to the WT, the two strains were compared using a multistep growth curve. This revealed that both strains exhibited remarkably similar growth kinetics in vitro (Figure 2B, top panel). Multiple comparisons tests conducted at each individual time point revealed no statistically significant differences between the WT and LucGFP strains, with similar observations made in a comparison of viral plaque areas (Figure 2B, lower panel). Collectively these findings indicate that the presence and expression of the reporter genes in the LucGFP recombinant had no discernible impact on replication kinetics, with the LucGFP recombinant strain maintaining comparable fitness to its parental strain in cell culture.

Next, the pathogenicity exhibited by the LucGFP recombinant in vivo was compared to that of its parental WT strain. WT and LucGFP recombinant strains inoculated by IP injection to adult fish showed comparable virulence as revealed by quantification of morbidity and mortality (Figure 2C). The clinical signs associated with CyHV-2 disease were inducted by both strains, and the intensities of the clinical signs were comparable in the two infection groups. Specifically, at 4 dpi, fish populations began to show recognizable clinical symptoms, including apathy, redness on the cephalic part, congestion, and hyperemia on the gills. One-week post-infection, the incidence rate reached 100%. In both groups, morbidity lasted for approximately one week, after which the survivors recovered and returned to a healthy state. The onset of mortality coincided closely with the onset of morbidity. This began at 5 dpi, with the number of deaths subsequently increasing rapidly. Mortality rate ceased approximately two weeks after infection. No significant difference in survival curves was detected between the WT and LucGFP groups. PCR assays targeting the LucGFP insert (Table 1) were performed on dead fish from the LucGFP group in order to exclude the possibility of contamination with the WT strain, and these confirmed the absence of contamination. These results indicated that the LucGFP recombinant exhibits comparable pathogenicity to that of its parental WT strain in vivo upon challenge by IP injection. Consequently, the LucGFP recombinant was deemed suitable to use in subsequent experiments to systematically investigate the spatiotemporal aspects of CyHV-2 replication in goldfish across larvae, juvenile, and adult developmental stages, using various in vivo imaging techniques.

### 3.3. Goldfish Larvae Are Susceptible and Highly Permissive to CyHV-2 Infection after Inoculation by Immersion in Infectious Water with the Skin of the Larvae Acting as the Portal of Entry of the Virus

**Figure 3:**
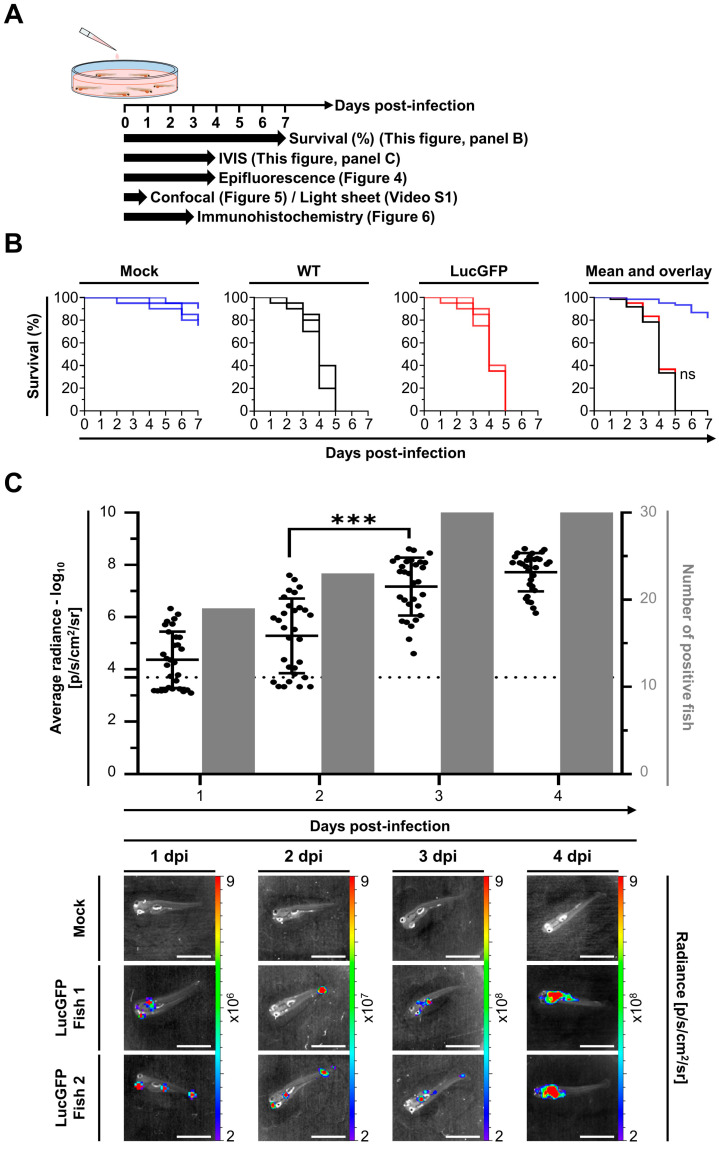
**Susceptibility and permissivity of goldfish larvae to CyHV-2.** (**A**) Flowchart of the experiments performed to investigate the susceptibility and permissivity of goldfish larvae (4 dpf or 1 d post-hatching, average length = 5.2 ± 0.1 mm) to CyHV-2 after infection by immersion in water containing the virus. (**B**) Survival curves of larvae following infection with the indicated strain. On day zero, 3 independent replicates of larvae, each group consisting of 60 subjects, were infected by immersion in E3 media containing the virus. Fish were examined daily, and those reaching the endpoints were euthanized. The percentage survival is expressed according to dpi. The three left panels show the survival curves observed for replicates. The right panel shows the mean survival curves based on the three replicates. (**C**) **Top** panel: Quantitative measurements of CyHV-2 replication in goldfish larvae obtained by IVIS. On day zero, three independent replicates of larvae, each group consisting of 50 subjects, were infected by immersion in E3 media containing the virus (5 × 10^5^ pfu/mL). At the indicated dpi, larvae (*n* = 30, i.e., 10 per replicate) were analyzed by IVIS. The average radiance (p/sec/cm^2^/sr) emitted by individual infected larvae corrected for the background of each image is represented by dots. For each time point, a group of mock-infected larvae was analyzed to define the threshold of positivity (dotted line), defined as the mean +3 SD. The number of positive larvae among 30 analyzed infected larvae is presented by grey bars. **Lower** panel: Representative images of analyzed larvae are presented in the lower part of the figure. Images are presented with a relative photon flux scale manually adapted to use the full dynamic range of the pseudo-color scale. Scale bar = 3 mm.

The LucGFP strain was used to establish the susceptibility and permissivity of goldfish larvae to CyHV-2. Larvae were challenged by immersion in infectious water, with survival and spatiotemporal aspects of infection subsequently examined using various methods. The experiment and designs are illustrated in Figure 3A. CyHV-2 challenge of larvae (4 dpf) by immersion resulted in 100% mortality in both WT and LucGFP infected groups by 5 dpi (Figure 3B). Most mortality occurred between 3–5 dpi and, consistent with Figure 2C (adult subjects, challenged by IP injection), there was no significant difference between WT and LucGFP survival curves (Figure 3B). In contrast, there was much less mortality in mock-infected groups, with 80% of subjects surviving up to 7 dpi.

Notably, the exceptionally high mortality observed (Figure 3B) suggested that goldfish larvae are highly susceptible to CyHV-2 infection, and possibly highly permissive to CyHV-2 replication once infections are established. The replication in larvae over time was subsequently investigated by using an IVIS to detect Luc expression after infection with the LucGFP strain, thus, facilitating relative quantitative analysis of viral load. This experiment also permitted the estimation of susceptibility, revealing that 60% of the population were positive for CyHV-2 (i.e., Luc expression) at 1 dpi, the earliest sampling point, with prevalence increasing to 100% by 3 dpi. The evolution of Luc expression over time (representing viral load) revealed that time post-infection had a significant impact on viral load (*p* < 0.0001). Viral load increased dramatically from 1–4 dpi, particularly after 3 dpi, with a significant increase in viral load observed between 2–3 dpi (Figure 3C, top panel), and was consistent with earlier observations indicating the onset of mortality peak at 3 dpi (Figure 3B). This indicated that goldfish larvae are highly permissive to CyHV-2 replication. This pattern was also reflected in a series of representative images from this analysis, showing that localized Luc signals, representing viral replication foci, were initially small and dispersed on larvae (1–3 dpi), but subsequently grew and coalesced into larger patches of infected tissue after 3 dpi (Figure 3C, lower panel).

**Figure 4:**
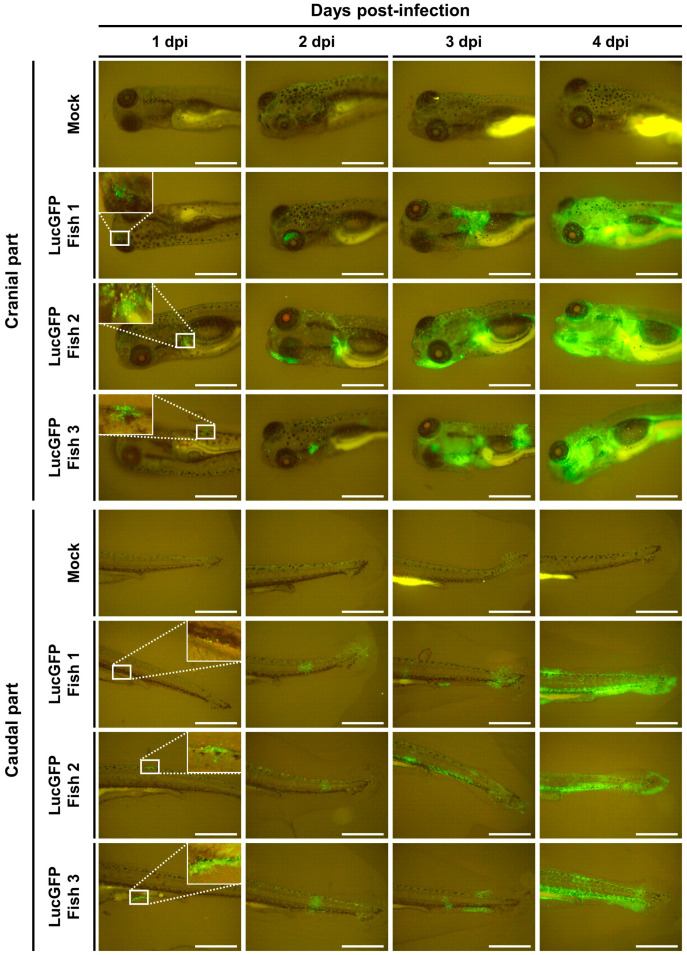
**Visualization of CyHV-2 infection in goldfish larvae using epifluorescence microscopy.** The timeline of this experiment has been described in Figure 3A. Epifluorescence microscopy images representative of larvae mock-infected and infected with the LucGFP strain according to time post-infection. Scale bar = 1 mm.

Identical infection experiments were conducted in parallel where CyHV-2 replication was monitored by epifluorescence microscopy, exploiting copGFP expression by the LucGFP strain. Consistent with the IVIS analysis (Figure 3C, top panel), this revealed an increase in copGFP signal with respect to time post-infection, in particular, after 3 dpi (Figure 4). Interestingly, careful microscopic examination of some subjects in different orientations suggested that the GFP signal may be entirely localized in the skin at 1 dpi (Figure 4, left panel), meaning that the skin may be the primary portal of entry of the virus into its host.

**Figure 5:**
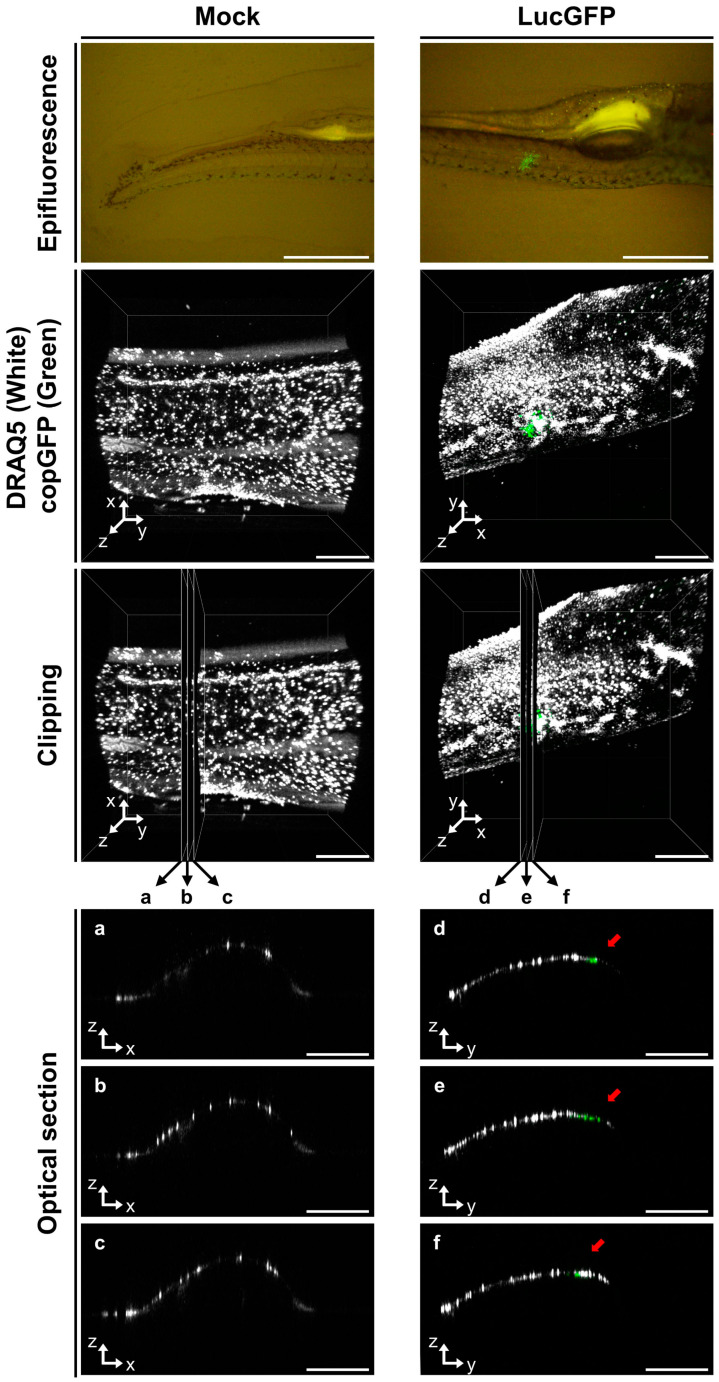
**Visualization of CyHV-2 infection in goldfish larvae using confocal microscopy.** The timeline of this experiment has been described in Figure 3A. The larvae were infected (LucGFP strain) or mock-infected by immersion in water containing the virus. At 1 dpi, fish were first observed by epifluorescence microscopy, and infected fish were identified based on the copGFP reporter signal (first row of panels, scale bar = 1 mm). Living skin epidermal cells on the outer body were then stained with DRAQ5. A series of Z-stacks were acquired using a confocal microscope in order to generate a 3D representation of the region of interest, with skin cells (DRAQ5-stained) in white and virally infected cells (copGFP expression) in green (second row of panel, scale bar = 300 µm). These data were used to generate three 2D optical sections in each sample (third row on panel), separated by a distance of 30 μm. These sections were denoted as a, b, and c for the mock-infected group, and d, e, and f for the LucGFP-infected group (each 0.662 μm in thickness). These optical sections are also displayed individually (three last rows, scale bar = 300 µm). Within the optical sections d, e, and f, it can be seen that the virally infected cells (copGFP green) only co-localize with skin epidermal cells (DRAQ5, white) and are indicated by red arrows. Conversely, no viral signal is detected in the mock-infected samples.

To investigate this further, in a more conclusive manner, we sought to selectively stain the epidermal skin cells in infected larvae and test for co-localization of epidermal and viral signal in 3D renderings of infected tissue. To achieve this, we first identified infected larvae via epifluorescence microscopy (Figure 5, first row), and exposed them to the DRAQ5 nuclear stain via addition to E3 media. The short DRAQ5 exposure and limited penetration during this period resulted in the selective staining of living epidermal skin cells in larval outer body. After this, fish were anesthetized and subjected to confocal microscopy to acquire Z-stacks from infected regions, which were subsequently used to generate 3D renderings (Figure 5, second row). In order to determine if the viral signals were localized in the skin only, and not originating from deeper tissue within the infected larvae, three optical sections intersecting with infected zones were generated from the 3D renderings (Figure 5, third row), which were examined further individually. These optical sections revealed co-localization of the copGFP and DRAQ5 signals in infected larvae (Figure 5, last three rows, optical sections d, e, and f,), thus, confirming that virally infected cells were restricted to the skin at 1 dpi. No viral signals were detected in a corresponding analysis of mock-infected fish (Figure 5, left panel).

In order to provide further support for these observations, rather than just focusing on specific regions of interest using confocal microscopy, 3D rendering was extended to entire larvae using light-sheet microscopy. In a similar experimental set-up, skin and virally infected cells in larvae (1 dpi) were again visualized using DRAQ5 live cell staining and copGFP expression, respectively. The findings from this experiment were consistent with the confocal analysis, indicating colocalization of viral signal in the skin only. A representative larval subject from this analysis is presented in Video S1, in which viral signal detected in the dorsal region is seen to be entirely localized in the skin.

**Figure 6:**
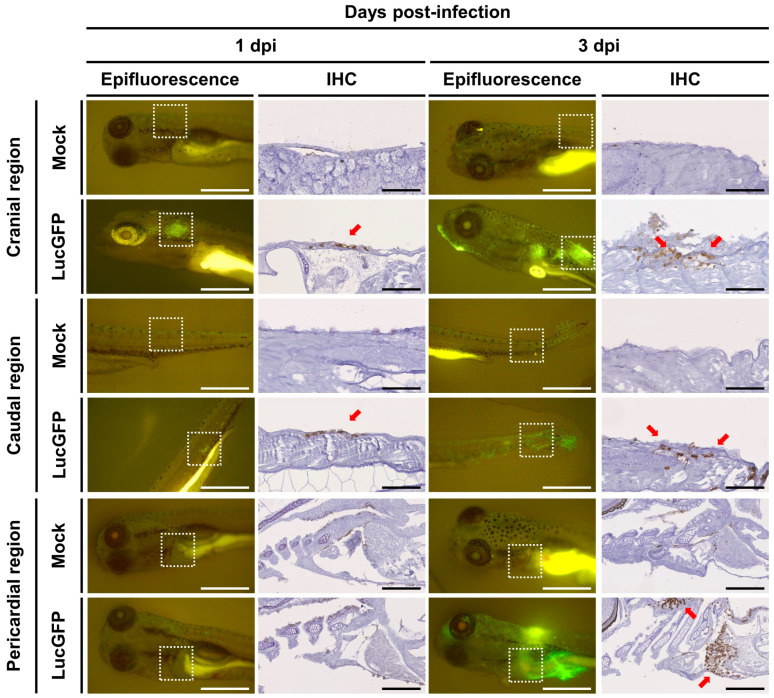
**Viral tropism of CyHV-2 in goldfish larvae.** The timeline of this experiment has been described in Figure 3A. Mock-infected and infected larvae (LucGFP strain) were first observed by epifluorescence microscopy (Epifluorescence, scale bar = 1 mm) at the indicated dpi, then processed for IHC detection of copGFP. The white boxes with dotted outlines in epifluorescence images indicate the regions analyzed by IHC. The virus was detected by staining for copGFP, with positive staining indicated by brown coloration filling entire cells (red arrows). Scale bars in IHC images represent 100 µm for anterior and caudal regions, and 200 µm for the pericardial region.

In parallel to these experiments, epifluorescence was used to select additional infected and mock-infected larvae (1 dpi), which were processed for analysis by IHC. This indicated that at 1 dpi, virus-infected cells were located primarily in the superficial layer of the skin at both cranial and caudal sites (Figure 6, left panel). Conversely, at the same time-point, the pericardial region exhibited a staining pattern that was comparable to the control group, with both being devoid of any viral staining, indicating the absence of systemic infection at this initial time point. Earlier IVIS analysis indicated that in infected larvae, a dramatic increase in viral load typically occurs after 3 dpi (Figure 3C). Consistent with this, IHC analysis of larvae sampled at 3 dpi revealed dramatic changes in viral distribution relative to observations at 1 dpi. By this timepoint, we observed that infection had breached the skin barrier and infiltrated other areas of the subject body, leading to the detection of CyHV-2 infected cells within the subcutaneous myotome and cardiac regions (Figure 6, right panel). These observations are likely to represent the onset of systemic infection and coincide with the expected onset of mortality peak at 3–5 dpi (Figure 3B).

Collectively, these findings provide compelling evidence that the portal of entry of CyHV-2 in goldfish larvae is the skin, and, from here, CyHV-2 spreads to other regions in larvae, leading to a systemic infection and ultimately to mortality.

### 3.4. Juvenile Goldfish Are Susceptible but Less Permissive than Goldfish Larvae to CyHV-2 Infection, with Initial Infection Predominantly Occurring in the Skin

**Figure 7:**
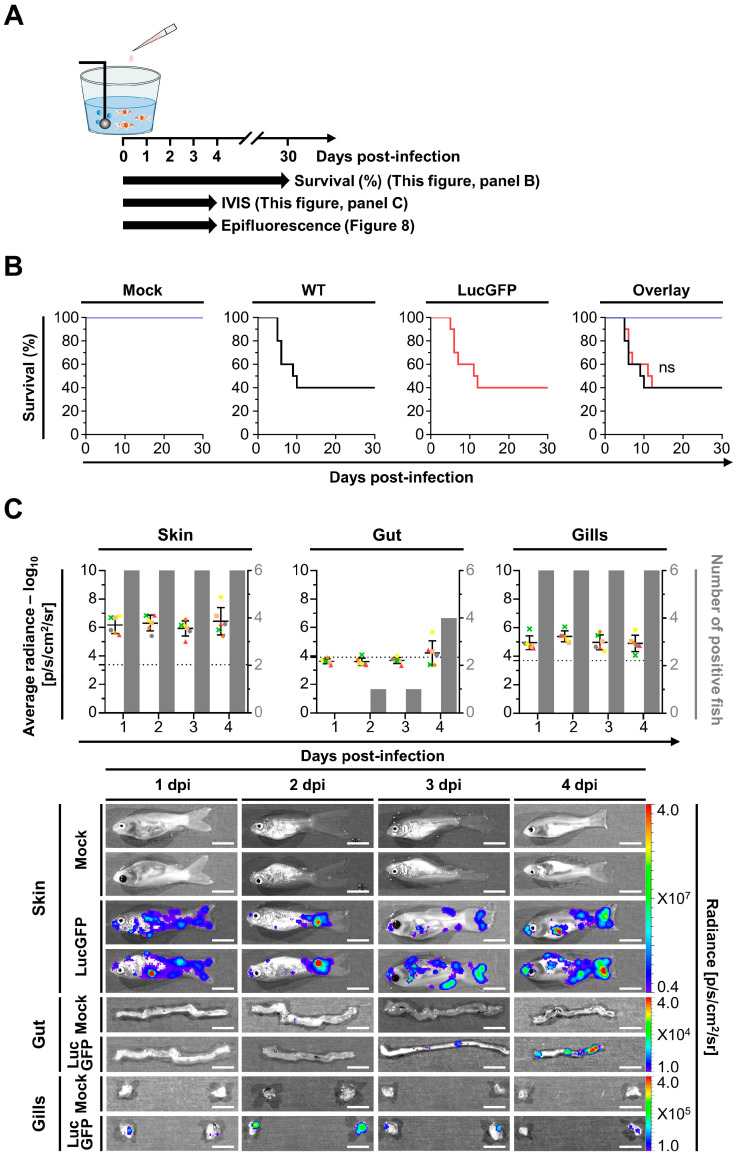
**Susceptibility and permissivity of juvenile goldfish to CyHV-2 infection.** (**A**) Flowchart of the experiments performed to investigate the susceptibility and permissivity of juvenile goldfish (75 days post fertilization, average length = 2.3 ± 0.3 cm) to CyHV-2 after infection by immersion in water containing the virus. (**B**) Survival rates of juveniles following infection with the indicated strain. On day zero, 10 subjects were infected by immersion in water containing the virus. Fish were examined daily and fish reaching the endpoints were euthanized. The percentage of survival is expressed according to dpi. The three left panels show the survival curves observed, respectively, for Mock, WT, and LucGFP groups. The right panel shows the overlay of the three groups. No statistically significant difference was observed between the WT and LucGFP strains. (**C**) **Top** panel: Quantitative measurements of CyHV-2 replication in juvenile goldfish by IVIS. Juveniles (*n* = 30) were infected (LucGFP strain) or mock-infected by immersion in infectious water. At the indicated dpi, juveniles (*n* = 6 per group) were analyzed by IVIS in vivo (skin) and ex vivo (gut and gills). The average radiance (p/sec/cm^2^/sr) emitted by individual infected juvenile corrected for the background of each image is represented by dots. For each time point, a group of mock-infected juvenile fish was analyzed to define the threshold of positivity (dotted line), defined as the mean +3 SD. The number of positive subjects among six analyzed infected juveniles is presented by grey bars. **Lower** panel: Representative images of analyzed juveniles are presented in the lower part of the figure. Images are presented with a relative photon flux scale manually adapted to use the full dynamic range of the pseudo-color scale. Scale bar = 5 mm.

The same LucGFP strain was also used to investigate the spatiotemporal aspects of CyHV-2 replication in juvenile goldfish. The experiment and designs are illustrated in Figure 7A. When subjected to challenge by immersion at the same dose used earlier with larvae, juvenile goldfish exhibited a survival rate of ~40% which was much higher than observed with larvae. Furthermore, mortality commenced at 5 dpi, which is later than in larvae, and continued until approximately 12 dpi, representing a much more gradual rate of mortality compared to larvae (Figure 7B). As per larvae, mortality rates were comparable between the WT and LucGFP infected groups, with no statistically significant difference observed.

Despite the lower mortality, in contrast to the larvae, the IVIS analysis in juvenile subjects revealed that all subjects were positive at 1 dpi and the viral load in the skin was much higher than in larvae at 1–2 dpi (Figure 7C, top panel). However, unlike in larvae, the viral load in the skin did not increase, with time post-infection having no significant impact on Luc signal. This developmental stage also facilitated ex vivo analysis of the gastrointestinal tract (gut) and gills. However, viral loads were extremely low in the gut, with no virus detected at 1 dpi, and virus detected in a single fish at 2 and 3 dpi. Notably, viral levels in the gut increased at 4 dpi (Figure 7C, top panel), which was immediately prior to the onset of mortality at 5 dpi (Figure 7B), but overall time post-infection had no impact on viral load in the gut. The prevalence and evolution of viral load in the gills was similar to the skin, with time having no significant impact on Luc signal; however, viral load in the gills was approximately 10-fold lower than in the skin (Figure 7C, top panel).

These patterns are also reflected in a series of representative images from IVIS analysis (Figure 7C, bottom panel). Notably the Luc signals from the skin are broadly distributed over a wide area. This pattern may have formed through coalescence of many initial infection foci. However, for this to be the case at 1 dpi, such coalescence would either have had to have occurred very rapidly, or there would need to have been a remarkably high initial number of infection foci. Both of these scenarios are consistent with the skin being more susceptible and permissive to CyHV-2 replication in juveniles, relative to other potential portals of entry. Conversely, signals from the gills tended to be more localized, originating from a singular source, with a similar distribution of viral signals in the gut, suggesting lower susceptibility or permissivity to CyHV-2 infection in these organs relative to the skin.

**Figure 8:**
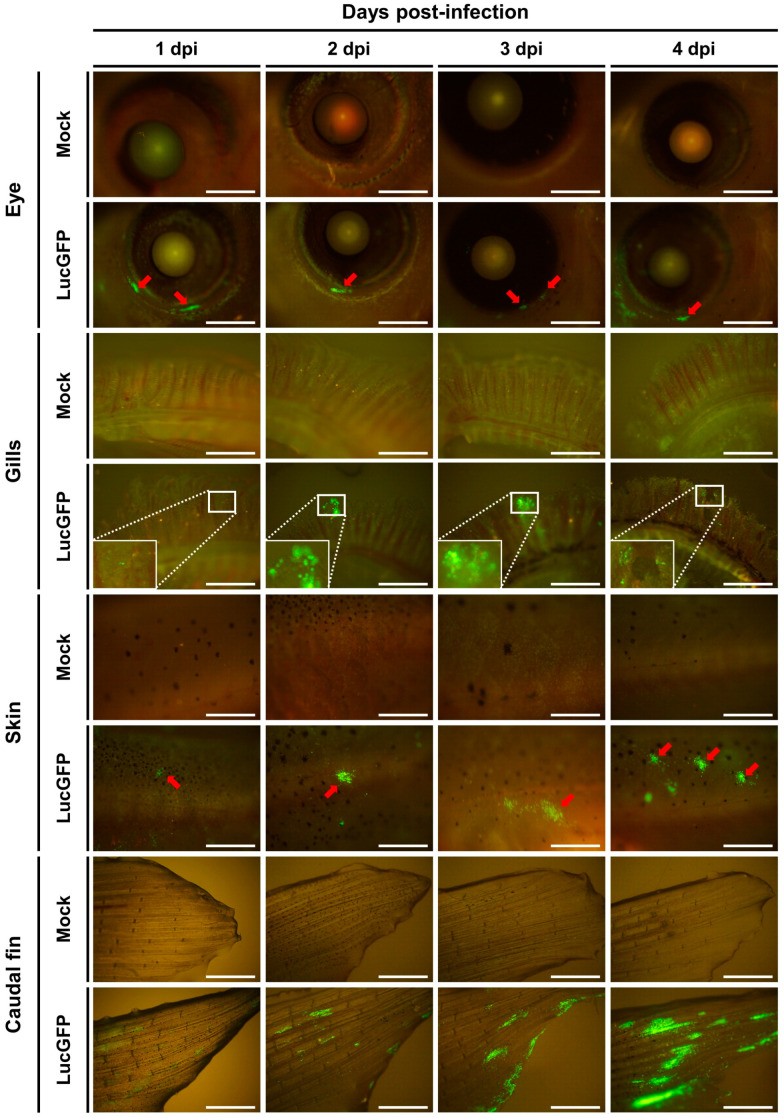
**Visualization of CyHV-2 infection in juvenile goldfish using epifluorescence microscopy.** The timeline of this experiment has been described in Figure 7A. Epifluorescence microscopy images representative of juvenile fish mock-infected and infected with the LucGFP strain according to time post-infection. Typical infection foci observed in the eye and skin regions are indicated with red arrows. Scale bars related to pictures of eye, skin, and caudal fin represent 1 mm. Scale bars related to pictures of gills represent 400 µm.

As per earlier larvae experiments, the expression of copGFP by the LucGFP strain facilitated further analysis of infected juveniles by epifluorescence microscopy (Figure 8). Four distinct areas exhibiting overt signs of infection were selected for further analysis, namely the eye, gill, skin, and caudal fin, with representative images displayed in Figure 8. Curiously, the edge of the cornea seemed to be particularly susceptible to infection. As indicated by the distribution of the Luc signal (Figure 7C, lower panel) viral infection foci on the skin and particularly in the caudal fin tended to be larger and proliferated slightly better over time, relative to other infection foci in the gills (Figure 8).

The three organs that were investigated in juveniles, namely skin, gills, and gut, represent the main mucosal surfaces in contact with the external environment, and, thus, should have equal contact with CyHV-2 during challenge by immersion. However, these results indicate that relative to other mucosal surfaces, the skin, being more susceptible and permissive, may play a much greater role in the entry of the virus into the host. Thus, the skin may represent the major portal of entry at this developmental stage, which is consistent with earlier observations in larvae.

The results from the juvenile stage provide an insight into changes in susceptibility and permissivity at an intermediate point in goldfish development after the larval stage but prior to the adult stage. On this note, given that CyHV-2 was detected in more juvenile subjects and at higher levels at 1 dpi, relative to what was observed in larvae, this may indicate that juveniles are in fact more susceptible than larvae. However, the lack of increase in viral load over time, coupled with the lower mortality, indicates that, overall, relative to larvae, the goldfish juvenile developmental stage may ultimately be less permissive to CyHV-2 replication.

### 3.5. Adult Goldfish Are Susceptible to CyHV-2 Infection, but Exhibit Lower Permissivity than Earlier Developmental Stages, with Initial Infection also Predominantly Occurring in the Skin

**Figure 9:**
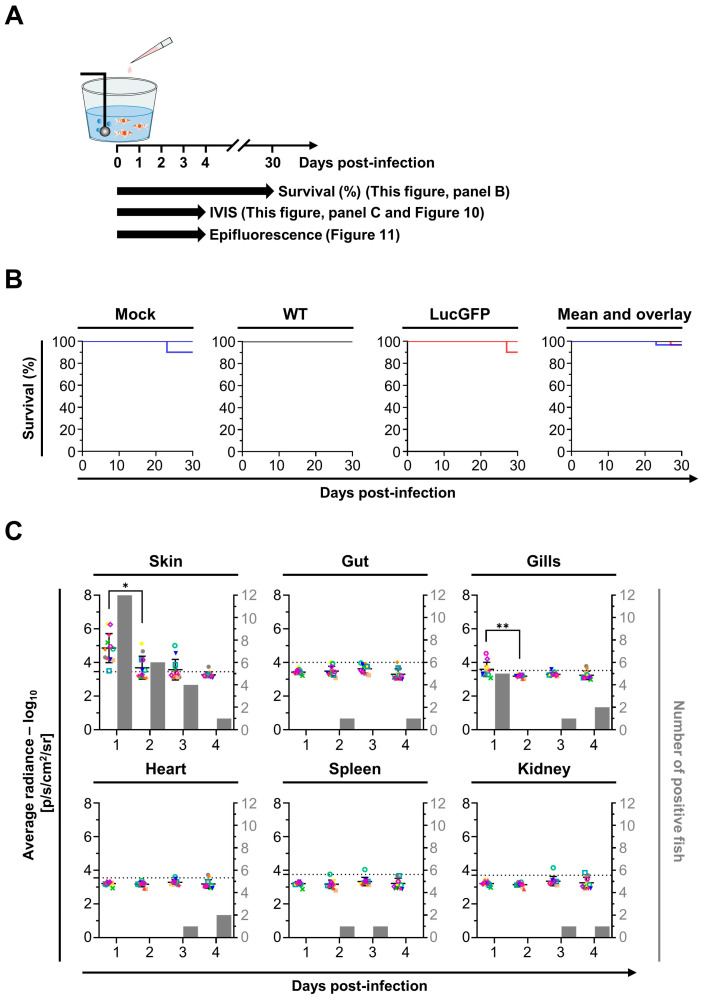
**Susceptibility and permissivity of adult goldfish to CyHV-2 infection.** (**A**) Flowchart of the experiments performed to investigate the susceptibility and permissivity of adult goldfish (1.5 years old, average weight = 12 ± 3.7 g) to CyHV-2 after infection by immersion in water containing the virus. (**B**) Survival rates of adults following infection with the indicated strain. On day zero, three independent replicates of adult fish (*n* = 30) were infected by immersion in water containing the virus. Fish were observed for 30 days and fish reaching the endpoints were euthanized. The percentage of survival is expressed according to dpi. The three left panels show the survival curves observed for replicates. The right panel shows the mean survival curves based on three replicates. (**C**) Quantitative measurements of CyHV-2 replication in adult goldfish by IVIS. Fish were infected or mock-infected with the LucGFP strain by immersion in infectious water. At the indicated dpi, fish (*n* = 12 per group) were analyzed by IVIS in vivo (skin) and ex vivo (gut, gills, heart, spleen, and kidney). The average radiance (p/sec/cm^2^/sr) emitted by individual infected fish corrected for the background of each image is represented by dots. For each time point, a group of mock-infected fish was analyzed to define the threshold of positivity (dotted line), defined as the mean +3 SD. The number of positive fish among 12 analyzed infected fish is presented by grey bars.

The spatiotemporal aspects of CyHV-2 infection in adults were also investigated, with experiments and designs illustrated in Figure 9A. In contrast to earlier developmental stages, adult goldfish demonstrated remarkably low mortality following challenge with CyHV-2 LucGFP and WT strains by immersion, with an almost 100% survival rate at 30 dpi (Figure 9B). This indicated that this goldfish developmental stage exhibits reduced permissivity to CyHV-2 replication, relative to earlier developmental stages. However, like the juvenile stage, all subjects were found to be infected at 1 dpi (Figure 9C). Viral load was also highest in the skin at 1 dpi compared to other organs, which was also consistent with the observations in juvenile goldfish. While the viral load was lower than the levels observed in the skin of juveniles at 1 dpi, it was higher than the levels observed in larvae at the same time point, indicating that adult goldfish are still relatively susceptible to CyHV-2 infection.

Being at a more advanced developmental stage, adult subjects also facilitated the ex vivo analysis of more internal organs, with heart, spleen, and kidney being added to the panel for this experiment. However, there was little viral signal detected in any internal organs across all timepoints (Figure 9C), indicating the absence of a low level of systemic infection. While skin and, to a lesser extent, gills, were more susceptible to infection than internal organs, a general decrease in viral load occurred in both of these organs after 1 dpi, with time post-infection having a significant negative impact on viral load (skin: *p* = 0.0002, gills: *p* = 0.0025), leading to virus replication being undetectable in most subjects by 4 dpi. This indicates that, while susceptible, adult goldfish were much less permissive to CyHV-2 infections relative to earlier developmental stages, and this is consistent with the extremely low mortality rate that was observed.

**Figure 10:**
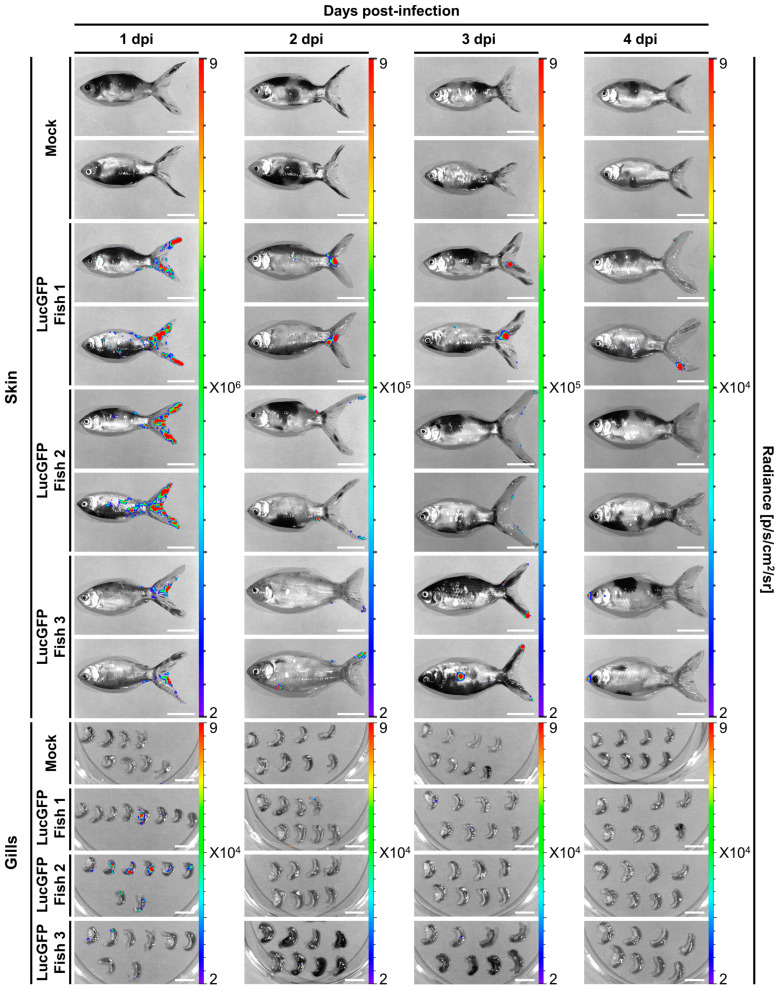
**Illustration of CyHV-2 tropism detected by IVIS in adult fish.** This figure is related to the experiment described in Figure 9C. Representative images of IVIS analysis are presented for skin and gills. Images are presented with a relative photon flux scale manually adapted in order to use the full dynamic range of the pseudo-color scale. Scale bars in panels related to skin and gills represent 2 and 1 cm, respectively.

**Figure 11:**
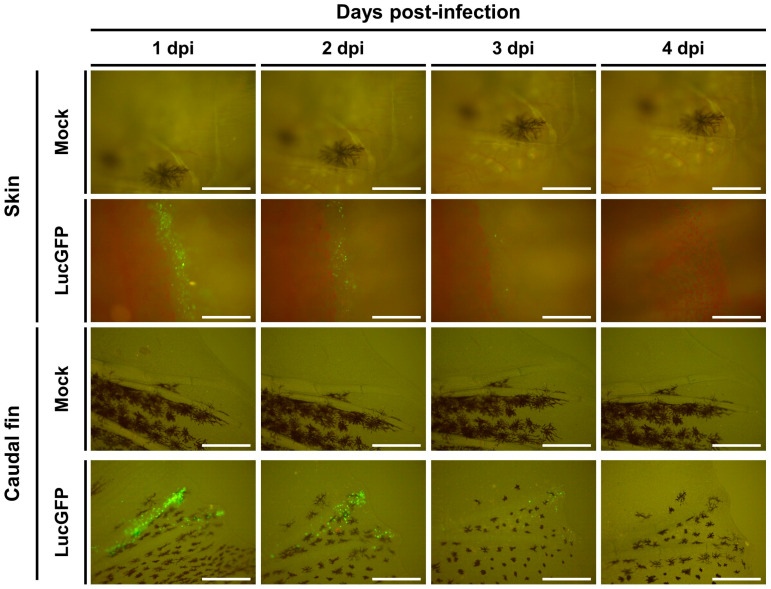
**Visualization of CyHV-2 infection in adult goldfish using epifluorescence microscopy.** This figure is related to the experiment described in Figure 9C. Epifluorescence microscopy images representative of adult fish mock-infected and infected with LucGFP strain according to time post-infection. Scale bar = 1 mm.

Interestingly, examination of the Luc signal distribution in skin and gills revealed that the caudal fin was particularly susceptible or possibly more permissive to infection at 1 dpi (Figure 10). A similar pattern was observed in further analysis by epifluorescence microscopy, with more signals again observed in the caudal fin relative to elsewhere on the skin (Figure 11). This is consistent with observations in juvenile subjects, where the caudal fin also exhibited a higher viral load (Figure 7C, lower panel and Figure 8). Taken together these results indicate that adult goldfish are much less permissive to CyHV-2 replication, compared to earlier developmental stages. However, consistent with other developmental stages, relative to other organs tested, the skin exhibited higher viral loads at earlier timepoints, indicating that it may naturally act as the major portal for CyHV-2 entry in populations of goldfish adults.

## 4. Discussion

In the present study, we developed a novel CyHV-2 recombinant strain expressing luciferase and GFP reporters. We subsequently successfully exploited this recombinant in the first investigation of the spatiotemporal aspects of CyHV-2 replication in goldfish across three key developmental stages. This facilitated the comparison of susceptibility and permissivity between different developmental stages and the identification of the CyHV-2 portal of entry in its natural host.

A crucial starting point for this study was the development of the CyHV-2 recombinant strain. Our initial systematic comparison of this new recombinant strain to the parental YC-01 strain revealed no difference in terms of expected genome structure (Figure 1B), expression of genes flanking the insertion site (Figure 1C), replication in cell culture (Figure 2B), and virulence by IP challenge (Figure 2C). Taken together, these results indicate that the LucGFP recombinant produced exhibits adequate properties for the study of CyHV-2 pathogenesis. This new recombinant is the first of its kind in the field of CyHV-2 research. We have previously developed a comparable recombinant to utilize in the study of CyHV-3, which is closely related to CyHV-2 and infects common carp, a species related to goldfish. The use of these earlier CyHV-3 recombinants had a profound impact on our ability to gain an increased fundamental understanding of this related virus and the development of adequate mitigation strategies [39,41,42,46,47,48,49]. In a similar manner, this novel CyHV-2 recombinant also represents a potentially valuable research tool for our future investigations with CyHV-2 and may also be of interest to the wider research community. Furthermore, in parallel to this present study, this novel CyHV-2 recombinant was also successfully utilized in zebrafish infectivity studies [45], demonstrating its immediate utility in other CyHV-2 related research.

One of the primary aims of this present study was to compare the susceptibility and permissivity of goldfish to CyHV-2 across the three distinct developmental stages: larvae, juvenile, and adults. This revealed that all stages were susceptible to infection (Figure 3C, Figure 7C and Figure 9C). Notably, infection was not as prevalent among larval subjects at the early time points post-infection by immersion and they also exhibited lower average radiance (representing viral load) at these time points, indicating reduced susceptibility. Despite this, larvae were still much more permissive to CyHV-2 replication, exhibiting the greatest increase in viral load over time, ultimately exceeding that of other developmental stages by 4 dpi, leading to much greater mortality (Figure 3B,C). In contrast, while the virus was detected in all juvenile hosts (indicating greater susceptibility) and the viral load was initially higher than in larvae, these levels remained stable over time (Figure 7C), resulting in reduced mortality in juveniles relative to the larval stage (Figure 7B). Finally, despite similarly high susceptibility, the adult stage was observed to be the least permissive to CyHV-2 replication with a decrease in viral load with respect to time (Figure 9C), and lower mortality relative to other developmental stages (Figure 9B).

The low mortality observed in this population of adult Shubunkin goldfish after CyHV-2 challenge by immersion (Figure 9B) relative to higher mortality by IP challenge (Figure 2C), is similar to previous findings involving comparisons between other breeds of goldfish [52]. However, given that Shubunkin goldfish are known to exhibit high mortality via natural infection routes [23], our observations may be specific to the population of Shubunkin goldfish that we used. It is highly likely that these contrasting phenotypes between different populations of Shubunkin goldfish may be due to genetic or epigenetic factors, which can have a significant impact on disease resistance in teleost fish hosts [53,54,55], including impacts on innate immune response to viral infection in cyprinid hosts [56]. Despite the lower mortality, challenge by immersion is much more representative of natural infection and had no impact on susceptibility to CyHV-2 among adults or juveniles in this study, which remained at 100%.

Interestingly, while viral prevalence was initially lower among larvae, it also increased with respect to time (Figure 3C). However, it is unclear if this was due to active viral transmission or simply due to the general increase in viral load, which may have been below the threshold for detection at earlier time points, leading to underestimation of initial infection prevalence. Notably, both of these scenarios are consistent with lower susceptibility to CyHV-2 at the larval stage in goldfish. Furthermore, this is consistent with our previous findings regarding susceptibility with CyHV-3 infection in carp [41], which represents the most closely related virus–host model to the CyHV-2-goldfish model [50,51]. The CyHV-3-carp model revealed that earlier carp developmental stages were less susceptible to CyHV-3 replication [41]. This reduced susceptibility was primarily due to the epidermal mucus being more inhibitory to CyHV-3 entry into hosts during earlier developmental stages. In addition to acting as a physical barrier, teleost epidermal mucus contains a multitude of antimicrobial proteins including proteases, immunoglobulins, complement components and innate immune cells that collectively inhibit viral infection at all developmental stages [57,58,59]. However, it is possible that the profiles of antimicrobial agents or physical uniformity of this protective barrier may change during development, as we discussed previously [41]. As observed in carp, it is possible that the epidermal mucus barrier in goldfish also plays a much greater inhibitory role at earlier developmental stages, resulting in lower CyHV-2 viral loads in larvae at earlier time points post CyHV-2 challenge by immersion. Currently, it is unclear which CyHV-2 factors may be responsible for providing some kind of resistance to this innate barrier in goldfish, leading to greater susceptibility at later developmental stages. However, we have recently demonstrated that in CyHV-3, mutations in ORF131 (which we previously determined to be a viral transmembrane protein [60]) have a significant impact on CyHV-3 resistance to innate immune components of carp epidermal mucus [48], although the implications at different developmental stages have yet to be determined. Notably, CyHV-2 and CyHV-3 share 19 homologous transmembrane proteins, including ORF131 [61]. It is plausible that this ORF131 homolog in CyHV-2 also plays a role in helping the virus overcome goldfish mucosal defenses, with potentially more efficacy at later developmental stages, thus, contributing to an initial greater viral load and prevalence among subjects at these later developmental stages. A combined investigation into the contribution of both the goldfish mucosal barrier and specific CyHV-2 membrane proteins at different developmental stages represents an interesting avenue for further investigation in the future, and may reveal factors impacting susceptibility, that are, evidently, independent from permissivity.

Despite potential differences in susceptibility, our observations indicate that the main factors dictating CyHV-2 related mortality at each developmental stage are differences in permissivity to CyHV-2 replication. Earlier goldfish developmental stages were much more permissive, leading to more mortality, despite being less susceptible to infection. This is in stark contrast to what we observed previously using the related CyHV-3-carp model, where in addition to being less susceptible, earlier carp developmental stages were also less permissive to CyHV-3 replication relative to later stages, which was accompanied by reduced mortality at earlier developmental stages [41]. This difference between CyHV-2 and CyHV-3 is surprising given how closely related they are [51,52]; however, this may be a consequence of differing adaptation to their respective hosts, or host-habitat. For example, unlike CyHV-3, CyHV-2 exhibits vertical transmission (i.e., from parent to offspring), and we hypothesize that this may be directly connected to differences between these two models in terms of permissivity at early developmental stages. Initial evidence for vertical transmission of CyHV-2 emerged in 2009 with the detection of CyHV-2 in disinfected eggs from infected goldfish [35]. Further investigation into this elsewhere led to the observation of viral particles in eggs of infected fish via electron microscopy and even the detection of active CyHV-2 gene transcription in eggs via RT-PCR [62]. Increased social contact between individuals during spawning potentially represents major mechanism for viral transmission among some fish species [50]. Given that (i) spawning is replaced by artificial reproduction practices in aquaculture settings and (ii) larger brood-stock may be subject to much lower stocking densities, and separated from the rest of the population, this may create selective pressure favoring viral strains capable of vertical transmission. However, it remains unclear if vertical transmission by CyHV-2 represents an adaptation to goldfish rearing practices, or if it emerged independently. Moreover, faced with potentially similar selective pressure associated with carp domestication, it is unclear why CyHV-3 does not also exhibit vertical transmission. However, recent phylogenetic dating studies that we conducted using different node calibration hypotheses consistently indicated that the CyHV-2 species clade emerged much more recently than CyHV-3, and also cast doubt on the plausibility that CyHV-3 emerged after the commencement of human aquaculture activities [50], which may explain these differences. However, such dating estimates need be supported by further investigation including collaborative efforts towards generating short-term evolutionary rate estimates for these viral species.

Regardless of the reasons why CyHV-2 evolved to exhibit vertical transmission, at a fundamental level, for it to occur, it is essential that extremely early developmental stages (−1 to 3 dpf) are permissive to CyHV-2 replication. More specifically, CyHV-2 must be capable of replicating in the populations of omnipotent, pluripotent, and rapidly dividing cells and undifferentiated tissue, which are in abundance at these stages. Such conditions may largely persist through the larval and, to some extent, during the juvenile stages investigated in this study (4 dpf and 75 dpf, respectively), and ultimately to a lesser extent as development continues, accompanied by further development of innate immune responses. While no other members of the genus *Cyprinivirus* are known to exhibit vertical transmission [50], in other more distantly related members of the family *Alloherpesviridae* that do, similar patterns can be observed across developmental stages. For example, Ictalurid herpesvirus 1 (IcHV-1, or Channel catfish virus) can also spread via vertical transmission, and, similarly to CyHV-2, disease caused by IcHV-1 is also primarily associated with earlier developmental stages [12,63,64]. Thus, it stands to reason that the ability of these alloherpesviruses to undergo vertical transmission may, as a natural consequence, also result in greater host permissivity at earlier developmental stages. However, this hypothesis needs to be further explored through more in-depth comparative virology across a wider range of relevant teleost virus–host models.

Another important aspect of the present study was to identify the portal of entry of the virus into the host. Previously it has been hypothesized that either the skin or gill could represent the portal of CyHV-2 entry in goldfish [20]. Others have speculated that in gibel carp, the gut represents a CyHV-2 portal of entry, supporting potential future oral vaccination strategies. Here, we compared the CyHV-2 viral loads during infection in different tissues in larval, juvenile, and adult developmental stages at 1, 2, and 3 dpi (and 4 dpi for the latter two). Unlike previous investigations, the experimental approach in this present study involved using a recombinant CyHV-2 strain expressing a reporter. The levels of this recombinant could be measured by IVIS and compared between different organs. As viral load can vary greatly between different regions of the skin, conducting such comparisons using other viral quantification approaches (e.g., PCR, ELISA, etc.) was not practical due to the inherent difficulty in identifying exactly which areas of the skin should be sampled prior to measurement. The use of the novel recombinant in this study was a key factor in facilitating non-biased measurements of viral load across the entire skin surface, thus, allowing valid comparisons to other organs.

In all developmental stages, we observed higher viral load in the skin at 1 dpi, the earliest sampling point post-infection, indicating that the skin represents a highly susceptible and the most permissive organ at early stages of infection after challenge by immersion. For example, in juvenile and adult subjects, viral load was higher in the skin than other organs, remaining so throughout the monitoring period (Figure 7C and Figure 9C). Furthermore, in adults and juveniles, the main source of signal in the skin was on the caudal fin, indicating that this region may be more susceptible to infection, and, hence, may act as the main portal of entry at these developmental stages. However, further comparison will be required to establish if this may in fact be related to the specific physiology of the Shubunkin goldfish caudal fin, rather than being the case for all goldfish breeds. While IVIS analysis was suitable for monitoring viral load over time at the larval stage allowing us to establish susceptibility and permissivity, given the constraints due to subject size, unlike the juvenile and adult stages, comparison between the skin and other organs in larvae, was not possible using this method. However, by selectively staining epidermal cells in the skin of larval subjects, we were able to confirm exclusive viral localization in this tissue at the earliest time point post-infection using 3D renderings generated from confocal (Figure 5) and light-sheet microscopy (Video S1). Furthermore, we established through histopathology that, in larvae, this initial infection on the skin was followed by spread to internal organs (Figure 6). Taken together, this indicates unequivocally that the skin represents the major portal of entry for the establishment of CyHV-2 infection in goldfish hosts. Furthermore, these findings across earlier and later developmental stages in CyHV-2-goldfish are entirely consistent with our previous findings in the CyHV-3-carp model [41,42], indicating a common host entry strategy in these two closely related models.

## 5. Conclusions

The present work represents the first systematic study of CyHV-2 pathogenesis and comparison between three major host developmental stages. It revealed that in goldfish (i) earlier developmental stages are less susceptible but more permissive to CyHV-2 infection relative to later developmental stages, ultimately leading to higher mortality in the former, and (ii) the skin represents the major portal of viral entry into the host. Collectively these findings provide important fundamental insights in CyHV-2 pathogenesis in goldfish, with high relevance to CyHV-2 infection in closely related economically important fish species and other related virus-host models. Furthermore, this work has resulted in the generation of the first recombinant CyHV-2 strain, and we have demonstrated that it represents a powerful new research tool that may be of interest to the wider CyHV-2 research community.

## Figures and Tables

**Table 1:** Primers and templates used in this study.

Description	Primer Name	Sequence (5′-3′)	Coordinates on Target Template ^1^
**For synthesis of LucGFP recombination cassette ^2^**			
Left homologous region	ORF64 HR Fw	*GGCGGCCGCGGGAATTCGAT*TCACTATGTATTTTCCCGC	102946-102964
	ORF64 HR Rev	GCAGATCCTTATTACATCGTGTGTCATTTATTAAG	103421-103445
LucGFP cassette	LucGFP Fw	ACGATGTAATAAGGATCTGCGATCGCTC	N/A: Template generated via ligation of several synthetic dsDNA fragments
	LucGFP Rev	TAATATGAACCCAGACATGATAAGATACATTGATG	
Right homologous region	ORF66 HR Fw	TCATGTCTGGGTTCATATTATATATATTTTATTGACGACAATAAAACC	103446-103483
	ORF66 HR Rev	*GCCGCGAATTCACTAGTGAT*ACGCAGACATCAACCTCATC	103926-103945
**For transcriptional analysis**			
ORF64	ORF64 Fw	CTGCATTGACAACAAGAAACGC	102098-102119
	ORF64 Rev	AAGAAGGGTTGGAGTCTCGAGC	102622-102643
ORF66	ORF66 Fw	CGTACTCTACCATGGGATGC	103739-103758
	ORF66 Rev	GTGGACATCATCACACAGGC	104329-104348
ORF79	ORF79 Fw	TTTGTGCTCTCTGAATCGGAGC	123777-123798
	ORF79 Rev	GCTGGTGACTTCTTTGTAGC	124177-124196

^1^ Coordinates relate to CyHV-2 YC-01 strain, GenBank accession number: MN593216.1. ^2^ Underlined, bases corresponding to CyHV-2 genome sequence; italic, bases corresponding to the pGEMT vector sequence; non-italic and no underlining, bases corresponding to the LucGFP expression cassette sequence.

## Data Availability

All data were include in this manuscripts.

## Supplementary Materials

The following supporting information can be downloaded at: https://www.mdpi.com/1999-4915/15/8/1746/s1, Video S1: Visualization of CyHV-2 virus infection in larvae using light-sheet fluorescence microscopy. Shubunkin goldfish larvae were infected by immersion with CyHV-2 LucGFP-contaminated water as described in Section 2. At 24 h pi, infected larvae were examined using light-sheet fluorescence microscopy. Nuclei of live skin epidermal cells in the larvae were stained with DRAQ5 (white contour around the body) while cells infected with LucGFP strain express GFP (green). In this 3D representation, virally infected cells were detected in the dorsal region of the larvae, and were entirely localized in the skin, with no virus detected in the internal tissue, thus, consistent with the observations in Figure 5.
