# Correction: He et al. In Vivo Imaging Sheds Light on the Susceptibility and Permissivity of *Carassius auratus* to Cyprinid Herpesvirus 2 According to Developmental Stage. *Viruses* 2023, *15*, 1746

**DOI:** 10.3390/v15112205

**Published:** 2023-11-01

**Authors:** Bo He, Arun Sridhar, Cindy Streiff, Caroline Deketelaere, Haiyan Zhang, Yuan Gao, Yunlong Hu, Sebastien Pirotte, Natacha Delrez, Andrew J. Davison, Owen Donohoe, Alain F. C. Vanderplasschen

**Affiliations:** 1 Immunology-Vaccinology, Department of Infectious and Parasitic Diseases, Fundamental and Applied Research for Animals & Health (FARAH), Faculty of Veterinary Medicine, University of Liège, B-4000 Liège, Belgium; 2 MRC-University of Glasgow Centre for Virus Research, Glasgow G61 1QH, UK; 3 Bioscience Research Institute, Technological University of the Shannon, Athlone N37 HD68, Co. Westmeath, Ireland

## 1. Missing Citation

In the original publication [1], some citation errors were accidentally introduced to this article during the finalizing stage, and therefore these errors were not attributed to the authors. The authors state that the scientific conclusions are unaffected. This correction was approved by the Academic Editor. The original publication has also been updated. The errors and changes are described as follows:

“Hanson, L.; Doszpoly, A.; van Beurden, S.J.; de Oliveira Viadanna, P.H.; Waltzek, T. Alloherpesviruses of Fish. In *Aquaculture Virology*; Kibenge, F.S.B., Godoy, M.G., Eds.; Academic Press: San Diego, CA, USA, 2016; pp. 153–172, ISBN 978-0-12-801573-5.” was not cited. The citation should be listed as Reference 64, which has now been inserted under “Discussion”, in Paragraph Number 7, and reads as follows:

Regardless of the reasons why CyHV-2 evolved to exhibit vertical transmission, at a fundamental level, for it to occur, it is essential that extremely early developmental stages (−1 to 3 dpf) are permissive to CyHV-2 replication. More specifically, CyHV-2 must be capable of replicating in the populations of omnipotent, pluripotent, and rapidly dividing cells and undifferentiated tissue, which are in abundance at these stages. Such conditions may largely persist through the larval and, to some extent, during the juvenile stages investigated in this study (4 dpf and 75 dpf, respectively), and ultimately to a lesser extent as development continues, accompanied by further development of innate immune responses. While no other members of the genus *Cyprinivirus* are known to exhibit vertical transmission [50], in other more distantly related members of the family *Alloherpesviridae* that do, similar patterns can be observed across developmental stages. For example, Ictalurid herpesvirus 1 (IcHV-1, or Channel catfish virus) can also spread via vertical transmission, and, similarly to CyHV-2, disease caused by IcHV-1 is also primarily associated with earlier developmental stages [12,63,64]. Thus, it stands to reason that the ability of these alloherpesviruses to undergo vertical transmission may, as a natural consequence, also result in greater host permissivity at earlier developmental stages. However, this hypothesis needs to be further explored through more in-depth comparative virology across a wider range of relevant teleost virus–host models. 

## 2. Incorrect Citations

The reference citation number 47 was included twice. A correction has been made to the “Results” section, under Section “3.1. Generation of the CyHV-2 LucGFP Recombinant Strain” in Paragraph Number 1, which should read as follows:

We have previously demonstrated that in vivo imaging is a highly effective methodology for the comprehensive investigation into pathogenesis associated with CyHV-3 [39,41,42,46–49] which is closely related to CyHV-2 [50,51]. To facilitate in vivo imaging during CyHV-2 experiments, the YC-01 CyHV-2 strain (WT) was selected as the parental strain for generation of a recombinant expressing Luc and copGFP as reporter proteins, which we refer to as the LucGFP strain. The reporter cassette was inserted between ORF64 and ORF66 of the WT CyHV-2 genome, as depicted in Figure 1A. This insertion of the construct resulted in the introduction of a new SacI restriction site at position 107281 of the LucGFP genome, resulting in the expected loss of the 7.66 kb SacI restriction fragment in the parental WT strain and an appearance of two smaller 5.1 KB and 6.7 kb SacI restriction fragments in the LucGFP strain (Figure 1A). The expected pattern was observed in subsequent SacI RFLP analysis (Figure 1B), thus, confirming the correct integration of the reporter cassette, which was also confirmed by whole genome sequencing (data not shown). Furthermore, transcriptomic analysis of ORF64 and ORF66 genes during LucGFP replication in vitro confirmed that the reporter cassette did not prevent the expression of genes adjacent to the insert in the LucGFP strain (Figure 1C).

With the above correction, the reference citation numbers 47–63 have been changed to 48–64, respectively.
